# Demographic and policy-based differences in behaviors and attitudes towards driving after marijuana use: an analysis of the 2013–2017 Traffic Safety Culture Index

**DOI:** 10.1186/s13104-021-05643-3

**Published:** 2021-06-03

**Authors:** Marco H. Benedetti, Li Li, Lucas M. Neuroth, Kayleigh D. Humphries, Ashley Brooks-Russell, Motao Zhu

**Affiliations:** 1 The Center for Injury Research and Policy, Abigail Wexner Research Institute At Nationwide Children’s Hospital, 700 Children’s Drive, RB3-WB5217, Columbus, OH 43215 USA; 2 Division of Epidemiology, College of Public Health, The Ohio State University, 1841 Neil Avenue, Columbus, OH 43210 USA; 3 Department of Community and Behavioral Health, Colorado School of Public Health, 13001 E. 17th Place, Mail Stop B119, Aurora, CO 80045 USA; 4 Department of Pediatrics, College of Medicine, The Ohio State University, 370 W. 9th Avenue, Columbus, OH 43210 USA

**Keywords:** Cannabis, Marijuana use, Legislation, Drugs, Driving after marijuana use, Marijuana laws, Per-se laws

## Abstract

**Objective:**

Marijuana policies in the United States have become more permissive, motivating research on demographic and policy-based differences in behaviors and attitudes towards driving after marijuana use. The Traffic Safety Culture Index is an annual survey with national scope and multiple measures relevant to driving after marijuana use. We tabulated responses to questions about driving after marijuana use from the Traffic Safety Culture Index based on demographic factors, recreational and medical marijuana policies, and per-se marijuana laws.

**Results:**

Male, younger, lower-income, and lower-education respondents self-reported driving after marijuana use more than their demographic counterparts, more often reported such behavior to be personally acceptable, and exhibited lower support per-se laws. Drivers in states that legalized medical marijuana self-reported driving after marijuana use slightly more than drivers in states where both medical and recreational were illegal. Support for per-se laws was higher among those in states that legalized recreational marijuana and in states with per-se laws. Demographic differences in our outcomes were consistent and cohesive. On the other hand, we found no predominant pattern suggesting that those in states with liberal marijuana policies were more tolerant of driving after marijuana use.

**Supplementary Information:**

The online version contains supplementary material available at 10.1186/s13104-021-05643-3.

## Introduction

Marijuana policies in the United States (U.S.) are becoming increasingly permissive, and research on the effects of these policies on driving after marijuana use is inconclusive. Studies of crash or driving data suggest that, although permissive marijuana policies are associated with higher prevalence of tetrahydrocannabinol (THC) presence among drivers [1, 2], there is mixed evidence that these policies are associated with motor vehicle crashes [3–9]. Because THC presence is an imperfect proxy for impairment [10], driving data may be limited in clarifying what traffic safety risks, if any, are associated with permissive marijuana policies.

An alternative approach to study relationships between marijuana policies and driving after marijuana use is to use surveys that collect information on self-reported behaviors and attitudes. However, relatively few studies have used self-reported data for this purpose [11–13]. Previous studies have explored differences in self-reported driving after marijuana use or driving under the influence of marijuana based on demographic factors and the presence of per-se marijuana laws, which equate impaired driving to a threshold of detectable THC in one’s system [14, 15], however they did not consider recreational or medical marijuana policies (RM and MM, respectively). Still others have examined behaviors and attitudes towards marijuana use in association with RM and MM policies, but did not analyze driving after marijuana use [16–27].

As marijuana policies continue to become more permissive in the US, there is a need for continued exploration of both demographic and policy-based differences in behaviors and attitudes towards driving after marijuana use. This paper does so through descriptive analysis of the Traffic Safety Culture Index (TSCI), an annual national survey conducted by the AAA Foundation for Traffic Safety.

## Main text

### Materials and methods

#### Study sample

We analyzed annual administrations of TSCI from 2013–2017, which asked respondents about various dimensions of traffic safety, including driving after marijuana use [28]. Surveys were administered to subjects from KnowledgePanel, a nationally representative online research panel [29]. During the study period, KnowledgePanel comprised approximately 55,000 members, which were selected via stratified probability sampling of the U.S. Postal Service’s Delivery Sequence File. Sampled non-internet households were provided with a notebook and internet services, which mitigates sampling bias typically associated with online panels [29]. Members of KnowledgePanel were sampled to participate in client surveys like TSCI. The resulting data included a set of survey weights, which accounted for selection probability and non-response. Furthermore, the weights were adjusted to ensure that the weighted sample resembles the Census Bureau’s Current Population Survey with respect to age, gender, race/ethnicity, education, census region, urbanicity, household composition, and household income [28].

Between 2013 and 2017, approximately 3000 TSCI surveys were completed annually. Respondents aged 19 and older were contacted directly and had a response rate around 60%. Respondents aged 16–18 were contacted through their parents, and their response rate was typically under 30% [28]. Due to these differences in sampling strategy and response rates, we excluded respondents aged 16–18. Under this restriction, we analyzed data from 11,816 respondents.

#### Measures

We analyzed responses to the following questions:

1. In the past year, how often have you driven within 1 h of using marijuana? (never vs. at least once).
2. How acceptable do you, personally, feel it is for a driver to drive 1 h after using marijuana? (completely/somewhat acceptable vs. completely/somewhat unacceptable).
3. How strongly do you support or oppose having a law making it illegal to drive with more than a certain amount of marijuana in your system? (strongly/somewhat support vs. strongly/somewhat oppose).

For question (1), we only included respondents who reported driving in the last month.

We obtained policy data from the National Conference of State Legislatures [30] and LexisNexis. By comparing respondents’ survey date to the effective dates of their state’s RM and MM policies, we assigned respondents to one of three policy categories: (i) RM, MM legal; (ii) MM legal, RM illegal; and (iii) RM, MM illegal. We also assigned binary indicators of per-se marijuana policies to each respondent. Tables S1 and S2 in Additional file 1 provide the effective dates of states’ RM, MM, and per-se marijuana policies.

#### Statistical analysis

We computed survey-weighted cross-tabulations of responses to questions (1)-(3) by gender, race/ethnicity, household income, age, education, and marijuana policies. We treated non-response, which occurred in less than 2% of responses, as missing.

To test for significant between-group heterogeneity in responses, we computed *p-*values based on Rao-Scott ${\chi }^{2}$ tests [31]. We conducted 21 statistical tests and employed a Bonferroni-Holm correction to ensure that the overall Type I Error rate was 0.05.^1^ We also computed 95% confidence intervals for all estimates, which were not adjusted for multiple comparisons.

### Results

Table 1 presents survey-weighted cross-tabulations of questions (1)–(3) by demographic factors and policies. Overall, 5.0% (95% CI 4.5%, 5.4%) of drivers reported driving after marijuana use (Table 1). Only 9.7% (95% CI 9.1%, 10.3%) of respondents reported that driving after marijuana use was acceptable, and 82.6% (95% CI 81.8%, 83.4%) of respondents supported per-se marijuana laws.

**Table 1.** 2013–2017 TSCI estimated past-year driving within 1 h of marijuana use, personal acceptance of driving after marijuana use, and support for per-se laws

Variable	Level	Unweighted sample size^a^	Weighted percent of total sample (95% CI)	Drivers who self-reported driving within one hour of using marijuana at least once in the last year		Respondents who said driving within one hour of using marijuana is somewhat or completely acceptable		Respondents who somewhat or strongly support a per-se marijuana law	
				Weighted percent (95% CI)	Rao-Scott $${\chi }^{2}$$ p-value^b^	Weighted percent (95% CI)	Rao-Scott $${\chi }^{2}$$ p-value^b^	Weighted percent (95% CI)	Rao-Scott $${\chi }^{2}$$ p-value^b^
Full sample	NA	11,816	–	5.0 (4.5, 5.4)	–	9.7 (9.1, 10.3)	–	82.6 (81.8, 83.4)	–
Gender	Male	5753	48.1 (47.1, 49.2)	6.5 (5.8, 7.3)		11.1 (10.1, 12.0)		80.5 (79.2, 81.7)	
	Female	6063	51.9 (50.8, 52.9)	3.5 (2.9, 4.1)	** < 0.001**	8.4 (7.6, 9.3)	** < 0.001**	84.4 (83.4, 85.5)	** < 0.001**
Income	< $25,000	1888	17.3 (16.5, 18.1)	9.4 (7.6, 11.2)		13.8 (12.0, 15.7)		76.0 (73.7, 78.3)	
	$25,000-$49,999	2570	22.0 (21.1, 22.9)	5.7 (4.5, 6.8)		10.1 (8.8, 11.5)		81.6 (79.8, 83.4)	
	$50,000-$74,999	2,220	18.2 (17.5, 19.0)	4.3 (3.2, 5.3)		9.2 (7.8, 10.7)		83.9 (82.1, 85.7)	
	$75,000-$99,999	1708	14.6 (13.9, 15.4)	3.8 (2.7, 4.9)		9.3 (7.6, 11.0)		82.9 (80.8, 85.0)	
	$100,000 or more	3430	27.8 (26.9, 28.7)	3.5 (2.8, 4.2)	** < 0.001**	7.3 (6.3, 8.3)	** < 0.001**	86.2 (84.8, 87.5)	** < 0.001**
Race/ethnicity	Non-Hispanic white	8696	66.0 (65.0, 67.1)	4.3 (3.8, 4.9)		9.9 (9.1, 10.6)		83.1 (82.1, 84.0)	
	Non-Hispanic black	1047	11.5 (10.8, 12.3)	7.5 (5.5, 9.5)		11.5 (9.2, 13.7)		76.5 (73.5, 79.5)	
	Non-Hispanic other	409	5.0 (4.4, 5.5)	5.9 (3.1, 8.7)		9.3 (6.1, 12.5)		89.5 (86.3, 92.6)	
	Hispanic	1319	15.0 (14.1, 15.8)	5.2 (3.7, 6.7)		7.7 (6.0, 9.4)		83.9 (81.6, 86.2)	
	Non-Hispanic 2 + races	345	2.5 (2.2, 2.9)	10.1 (5.3, 14.8)	**0.002**	9.6 (6.1, 13.2)	0.319	74.6 (68.9, 80.3)	** < 0.001**
Education	Less than high school	1060	12.4 (11.6, 13.1)	8.5 (6.3, 10.7)		11.0 (8.9, 13.2)		78.1 (75.3, 80.9)	
	High school	3448	28.7 (27.7, 29.6)	4.7 (3.8, 5.5)		9.7 (8.6, 10.9)		82.0 (80.5, 83.5)	
	Some college	3389	29.7 (28.8, 30.7)	5.5 (4.5, 6.4)		11.1 (9.9, 12.4)		81.4 (79.9, 83.0)	
	Bachelor’s or higher	3919	29.2 (28.3, 30.2)	3.6 (2.9, 4.3)	** < 0.001**	7.6 (6.6, 8.6)	**0.002**	86.0 (84.7, 87.3)	** < 0.001**
Age	19–29	1870	19.7 (18.8, 20.6)	11.6 (9.7, 13.4)		15.1 (13.3, 17.0)		76.0 (73.7, 78.3)	
	30–44	2661	25.7 (24.7, 26.6)	5.6 (4.6, 6.5)		12.0 (10.6, 13.5)		79.8 (78.1, 81.6)	
	45–59	3608	27.3 (26.4, 28.2)	3.4 (2.7, 4.1)		8.8 (7.7, 9.8)		83.4 (82.0, 84.8)	
	60 or older	3677	27.4 (26.5, 28.3)	1.8 (1.3, 2.3)	** < 0.001**	4.6 (3.8, 5.4)	** < 0.001**	88.7 (87.5, 89.9)	** < 0.001**
Marijuana Policy	RM and MM illegal	6011	51.5 (50.4, 52.5)	4.3 (3.7, 5.0)		9.6 (8.7, 10.5)		81.7 (80.5, 82.8)	
	RM illegal, MM legal	4855	41.0 (40.0, 42.0)	5.9 (5.1, 6.8)		9.6 (8.6, 10.6)		82.6 (81.4, 83.9)	
	RM and MM legal	950	7.5 (7.0,8.1)	4.2 (2.7, 5.8)	**0.024**	10.6 (8.2,13.0)	0.733	87.8 (85.3, 90.3)	**0.003**
Per-se law	Absent	8769	72.8 (71.9, 73.8)	5.1 (4.6, 5.7)		10.0 (9.3, 10.8)		81.6 (80.6, 82.5)	
	Present	3047	27.2 (26.2, 28.1)	4.5 (3.6, 5.4)	0.563	8.8 (7.6, 9.9)	0.319	85.1 (83.6, 85.5)	**0.002**

^a^The unweighted sample size includes those with missing values or non-response to questions (1)–(3)

^b^Boldface denotes statistically significant between-group heterogeneity at the significance level of 0.05. *p*-values are adjusted using a Bonferroni-Holm correction

Male, younger, low-income, low education (less than high school), non-Hispanic black, and non-Hispanic multiracial drivers self-reported driving after marijuana use more than their counterparts. Similarly, male, younger, low-income, and low-education respondents exhibited higher acceptance of such behavior, and lower support for per-se marijuana laws. While non-Hispanic black and non-Hispanic multiracial respondents supported per-se marijuana laws less than their counterparts (*p* < 0.001), we found little evidence of between-race heterogeneity in personal acceptance of driving after marijuana use (*p* = 0.319).

A higher percentage of drivers in states that legalized MM but not RM self-reported driving after marijuana use, compared to drivers in states that had not legalized RM and MM (5.9% vs. 4.3%; *p* = 0.024). However, there was practically no difference in self-reported driving after marijuana use between drivers in states that legalized RM and drivers in states that had not (4.2% vs. 4.3%). The same was true comparing drivers in states with per-se marijuana laws to drivers in states with no such law (4.5% vs. 5.1%; *p* = 0.563). A higher percentage of respondents in states that legalized RM supported per-se laws, compared to those in states where RM was illegal (87.8% RM and MM Legal vs. 82.6% MM Legal vs. 81.3% RM and MM Illegal; *p* = 0.003). The same was true of those in states that had per-se laws compared to those in states that did not (85.1% vs. 81.6%; *p* = 0.002).

### Discussion

Our study analyzed a survey that is well-suited for drug policy research due to its national scope and measures related to driving after marijuana use. We identified multiple patterns in behaviors and attitudes towards driving after marijuana use based on demographic factors and policies.

We observed cohesion in responses to the three outcomes based on demographic factors. Younger, low-income, low-education, and male respondents (i) more often self-reported driving after marijuana use; (ii) more often found such behavior acceptable; and (iii) less often supported per-se marijuana laws than their counterparts. This pattern suggests that these demographic groups are, on average, more tolerant of driving after marijuana use. Our finding that a higher percentage of male and younger drivers self-reported driving after marijuana use was consistent with Azofeifa et al. who examined self-reported driving under the influence of marijuana using the National Survey on Drug Use and Health (NSDUH) [15]. However, compared to Azofeifa et al. we found greater heterogeneity in responses based on race/ethnicity. This may be because NSDUH asks respondents to judge whether they were impaired, whereas TSCI asks about driving within an hour of using marijuana.

Drivers in states that legalized MM self-reported driving after marijuana use more than their counterparts in states where MM was illegal, which was consistent with studies finding that MM legalization was associated with higher prevalence of THC detection in drivers [1, 2]. Fink et al. and Benedetti et al. both found evidence that MM policies were associated with higher self-reported driving after marijuana use [11, 12]. These findings are consistent with the policy-based difference in the present study; however we did not control for potential confounders or baseline differences in the outcome. Unlike Lensch et al. we did not find that self-reported driving after marijuana use is more prevalent in states that legalized RM [13]. A possible explanation for this discrepancy is the difference in policy dates used in either study: Lensch et al. used the date on which commercial sales of RM were legalized, whereas we used the effective dates of RM legalization.

Lensch et al. also found that marijuana users in states that legalized sales of marijuana had lower prevalence of self-reported driving after marijuana use and more protective attitudes about such behavior. Table S4 in Additional file 1 presents policy-based differences in the outcomes among respondents who self-reported marijuana use in the last year. Consistent with Lensch et al. marijuana users in states that legalized RM self-reported driving after marijuana use less than their counterparts. They were also less likely to find such behavior acceptable and more likely to support per-se laws.

Respondents in states that legalized RM exhibited higher support for per-se marijuana laws, perhaps due to a perception that RM legalization makes them more likely to encounter impaired drivers. Furthermore, if we interpret support for per-se laws as a protective behavior, then this result is consistent with Lensch et al., who found that people in states that legalized sales of RM were more likely to try to stop a friend from driving while drunk or high.

In contrast to demographic results, we observed no cohesive patterns in the three outcomes based on marijuana policy. Furthermore, we note that some statistically significant differences were of small magnitude and may not have been meaningful from a practical standpoint.

Based on low overall personal acceptance of driving after marijuana use and high support for per-se laws, this study suggests that the public widely perceives driving after marijuana use to be a dangerous behavior. The authors share this view, assessing that the current evidence shows that marijuana can impair driving ability and increase crash risk, especially shortly after consumption. However, we acknowledge that not all studies support this position [10, 32–34]. Experimental and driving simulator studies have shown that marijuana affects motor skills and executive function [35–39], but this need not correspond to increased crash risk. For instance, Lacey et al. found no evidence of an association between THC presence in drivers and the odds of fatal crash involvement [40]. Additional epidemiological studies have reported statistically significant associations between THC presence in drivers and higher odds of fatal crash involvement and causation, however estimated odds ratios, ranging from 1.17 to 1.62, were arguably small in magnitude [41–44]. THC can remain in one’s system long after cognitive effects are experienced, and tolerance varies greatly between users. Therefore, studies of crash data struggle to capture what, if any, impairment was experienced by the drivers at the time of the crash, especially when the time between consumption and crash is unknown. A meta-analysis by Asbridge et al. which only included studies of crash risk associated with recent marijuana use, found that THC presence was associated with significantly higher odds of motor vehicle collisions (OR 1.92; 95% CI 1.35, 2.73) [32].

### Conclusions

Based on self-reported behaviors and attitudes, certain demographic groups were more tolerant of driving after marijuana use than their counterparts. In contrast, we found no predominant pattern suggesting that behaviors and attitudes were more tolerant in states with liberal marijuana policies.

## Limitations

Our study had several limitations. First, all outcomes were self-reported. Although drug-impaired driving is illegal throughout US, respondents in states with strict marijuana policies may be less forthright about their behavior. Second, our study is entirely descriptive. The results herein may have been subject to confounding, and do not provide evidence for or against causal effects of marijuana policies. Third, we only considered three types of marijuana policies. There is evidence that accounting more specific marijuana policy dimensions can impact findings related to their effects [24]. Finally, our only available measure of driving exposure was a binary indicator of past-month driving.

## Supplementary Information

**Additional file 1. Supplementary Tables:** Tables S1 and S2 tabulate the effective dates of recreational, medical, and per-se marijuana policies that were used in the main analysis.  Table S3 presents the results from the main analysis, however the *p-*values are not corrected for multiple comparisons, as they were in the manuscript.  Table S4 presents policy-based differences in the three outcomes only among those who self-reported using marijuana in the last year.

## Data Availability

The datasets supporting the current study can be obtained by contacting the AAA Foundation for Traffic Safety and completing a request form.

## Footnotes

1 We considered both Bonferroni and Bonferroni-Holm corrections as candidate methods for adjusting the *p*-values. We preferred these adjustments over others (e.g. Benjamini–Hochberg or Holm-Šidák) because they did not require assumptions about dependence between hypotheses. We selected Bonferroni-Holm because it is uniformly more powerful than the classic Bonferroni adjustment. Table S3 in the Additional file 1 provides unadjusted *p*-values to allow adjustment using other methods.

## References

[1] Couper FJ, Peterson BL (2014). The prevalence of marijuana in suspected impaired driving cases in Washington State†. J Anal Toxicol.

[2] Johnson MB, Kelley-Baker T, Voas RB, Lacey JH (2012). The prevalence of cannabis-involved driving in California. Drug Alcohol Depend.

[3] Aydelotte JD, Brown LH, Luftman KM, Mardock AL, Teixeira PGR, Coopwood B (2017). Crash fatality rates after recreational marijuana legalization in Washington and Colorado. Am J Public Health.

[4] Aydelotte JD, Mardock AL, Mancheski CA, Quamar SM, Teixeira PG, Brown CVR (2019). Fatal crashes in the 5 years after recreational marijuana legalization in Colorado and Washington. Accident Anal Prev.

[5] Cook AC, Leung G, Smith RA (2020). Marijuana Decriminalization, Medical Marijuana Laws, and Fatal Traffic Crashes in US Cities, 2010–2017. Am J Public Health.

[6] Hansen B, Miller K, Weber C (2018). Early evidence on recreational marijuana legalization and traffic fatalities. Econ Inquiry..

[7] Kamer RS, Warshafsky S, Kamer GC (2020). Change in traffic fatality rates in the first 4 states to legalize recreational Marijuana. JAMA Inter Med.

[8] Santaella-Tenorio J, Mauro CM, Wall MM, Kim JH, Cerda M, Keyes KM (2017). US traffic fatalities, 1985–2014, and their relationship to medical Marijuana Laws. Am J Public Health.

[9] Santaella-Tenorio J, Wheeler-Martin K, DiMaggio CJ, Castillo-Carniglia A, Keyes KM, Hasin D (2020). Association of Recreational Cannabis Laws in Colorado and Washington State With Changes in Traffic Fatalities, 2005–2017. JAMA Inter Med.

[10] Hartman RL, Huestis MA (2013). Cannabis effects on driving skills. Clin Chem.

[11] Benedetti MH, Li L, Neuroth LM, Humphries KD, Brooks-Russell A, Zhu M (2020). Self-reported driving after marijuana use in association with medical and recreational marijuana policies. Int J Drug Policy..

[12] Fink DS, Stohl M, Sarvet AL, Cerda M, Keyes KM, Hasin DS (2020). Medical marijuana laws and driving under the influence of marijuana and alcohol. Addiction.

[13] Lensch T, Sloan K, Ausmus J, Pearson JL, Clements-Nolle K, Goodman S (2020). Cannabis use and driving under the influence: behaviors and attitudes by state-level legal sale of recreational cannabis. Prev Med.

[14] Arnold LS, Tefft BC (2016). Driving under the influence of alcohol and marijuana: beliefs and behaviors, United States, 2013–2015.

[15] Azofeifa A, Rexach-Guzmán BD, Hagemeyer AN, Rudd RA, Sauber-Schatz EK (2019). Driving under the influence of marijuana and illicit drugs among persons aged ≥16 years—United States, 2018. MMWR Morb Mortal Wkly Rep.

[16] Anderson DM, Hansen B, Rees DI, Sabia JJ (2019). Association of marijuana laws with teen marijuana use: new estimates from the youth risk behavior surveys. JAMA Pediatr.

[17] Cerdá M, Wall M, Feng T, Keyes KM, Sarvet A, Schulenberg J (2017). Association of state recreational marijuana laws with adolescent marijuana use. JAMA Pediatr.

[18] Hasin DS, Saha TD, Kerridge BT, Goldstein RB, Chou SP, Zhang H (2015). Prevalence of marijuana use disorders in the United States between 2001–2002 and 2012–2013. JAMA Psychiat.

[19] Hasin DS, Sarvet AL, Cerdá M, Keyes KM, Stohl M, Galea S (2017). US adult illicit cannabis use, cannabis use disorder, and medical marijuana laws: 1991–1992 to 2012–2013. JAMA Psychiat.

[20] Hasin DS, Wall M, Keyes KM, Cerdá M, Schulenberg J, O'Malley PM (2015). Medical marijuana laws and adolescent marijuana use in the USA from 1991 to 2014: results from annual, repeated cross-sectional surveys. Lancet Psychiatry.

[21] Khatapoush S, Hallfors D (2004). “Sending the Wrong Message”: did medical marijuana legalization in california change attitudes about and use of marijuana?. Journal of Drug Issues.

[22] Kosterman R, Bailey JA, Guttmannova K, Jones TM, Eisenberg N, Hill KG (2016). Marijuana legalization and parents' attitudes, use, and parenting in Washington State. J Adolesc Health.

[23] Miller A, Rosenman R, Cowan B (2017). Recreational marijuana legalization and college student use: Early evidence. SSM Popul Health.

[24] Pacula RL, Powell D, Heaton P, Sevigny EL (2015). Assessing the effects of medical marijuana laws on marijuana use: the devil is in the details. J Policy Anal Manage.

[25] Sarvet AL, Wall MM, Fink DS, Greene E, Le A, Boustead AE (2018). Medical marijuana laws and adolescent marijuana use in the United States: a systematic review and meta-analysis. Addiction.

[26] Smart R, Pacula RL (2019). Early evidence of the impact of cannabis legalization on cannabis use, cannabis use disorder, and the use of other substances: findings from state policy evaluations. Am J Drug Alcohol Abuse.

[27] Wen H, Hockenberry JM, Druss BG (2019). The effect of medical marijuana laws on marijuana-related attitude and perception among US adolescents and young adults. Prev Sci.

[28] AAA Foundation for Traffic Safety. 2017 Traffic Safety Culture Index: AAA Foundation for Traffic Safety; 2018. https://aaafoundation.org/2017-traffic-safety-culture-index/.

[29] GfK. GfK KnowledgePanel 2016. https://www.gfk.com/fileadmin/user_upload/dyna_content/US/documents/GfK_KnowledgePanel_Overview.pdf.

[30] National Conference of State Legislatures. State Medical Marijuana Laws: National Conference of State Legislatures; 2019. https://www.ncsl.org/research/health/state-medical-marijuana-laws.aspx.

[31] Rao JNK, Scott AJ (1987). On simple adjustments to chi-square tests with sample survey data. Ann Statist.

[32] Asbridge M, Hayden JA, Cartwright JL (2012). Acute cannabis consumption and motor vehicle collision risk: systematic review of observational studies and meta-analysis. BMJ..

[33] Sewell RA, Poling J, Sofuoglu M (2009). The effect of cannabis compared with alcohol on driving. Am J Addict.

[34] Aston ER, Merrill JE, McCarthy DM, Metrik J (2016). Risk Factors for Driving After and During Marijuana Use. J Stud Alcohol Drugs..

[35] Ramaekers JG, Kauert G, van Ruitenbeek P, Theunissen EL, Schneider E, Moeller MR (2006). High-potency marijuana impairs executive function and inhibitory motor control. Neuropsychopharmacology.

[36] Hollis C, Karoly MAM, Brooks-Russell A, Brown M, Streufert J, Bryan AD, Lovrich NP, DeJong W, Bidwell LC (2020). Effects of high-potency cannabis on psychomotor performance in frequent cannabis users. Cannabis Cannabinoid Res.

[37] Hartman RL, Brown TL, Milavetz G, Spurgin A, Pierce RS, Gorelick DA (2015). Cannabis effects on driving lateral control with and without alcohol. Drug Alcohol Depend.

[38] Ronen A, Gershon P, Drobiner H, Rabinovich A, Bar-Hamburger R, Mechoulam R (2008). Effects of THC on driving performance, physiological state and subjective feelings relative to alcohol. Accid Anal Prev.

[39] Arkell TR, Vinckenbosch F, Kevin RC, Theunissen EL, McGregor IS, Ramaekers JG (2020). Effect of cannabidiol and Δ9-tetrahydrocannabinol on driving performance: a randomized clinical trial. JAMA.

[40] Lacey JH, Berning A, Romano E, Ramirez A, Julie Yao CM, Brainard K, Carr K, Pell K, Compton R. Drug and alcohol crash risk: a case-control study (Report No. DOT HS 812 355). 2016.

[41] Li G, Chihuri S, Brady JE (2017). Role of alcohol and marijuana use in the initiation of fatal two-vehicle crashes. Ann Epidemiol.

[42] Chihuri S, Li G, Chen Q (2017). Interaction of marijuana and alcohol on fatal motor vehicle crash risk: a case–control study. Inj Epidemiol.

[43] Dubois S, Mullen N, Weaver B, Bédard M (2015). The combined effects of alcohol and cannabis on driving: Impact on crash risk. Forensic Sci Int.

[44] Bédard M, Dubois S, Weaver B (2007). The impact of Cannabis on driving. Can J Public Health.

