# Supplementary material for: Demographic and policy-based differences in behaviors and attitudes towards driving after marijuana use: an analysis of the 2013–2017 Traffic Safety Culture Index
Source: BMC Res Notes. 2021 Jun 3;14:226. doi: 10.1186/s13104-021-05643-3 (PMC8176701; doi:10.1186/s13104-021-05643-3)
Supplement: Supplementary file 1 — Additional file 1. Supplementary Tables: Tables S1 and S2 tabulate the effective dates of recreational, medical, and per-se marijuana policies that were used in the main analysis. Table S3 presents the results from the main analysis, however the p-values are not corrected for multiple comparisons, as they were in the manuscript. Table S4 presents policy-based differences in the three outcomes only among those who self-reported using marijuana in the last year. [file 13104_2021_5643_MOESM1_ESM.docx]

**Additional Materials for** ***Demographic and policy-based differences in behaviors and attitudes towards driving after marijuana use: an analysis of the 2013-2017 Traffic Safety Culture Index.***

Marco H. Benedetti^a^, Li Li^a,b^, Lucas M. Neuroth^a,b^, Kayleigh D. Humphries^a^, Ashley Brooks-Russell^c^, Motao Zhu­^a,b,d^

^a^The Center for Injury Research and Policy, Abigail Wexner Research Institute at Nationwide Children’s Hospital

700 Children’s Drive

Columbus, OH 43215

USA

^b^Division of Epidemiology, College of Public Health, The Ohio State University

1841 Neil Avenue
Columbus, OH 43210

USA

^c^Department of Community and Behavioral Health, Colorado School of Public Health

13001 E. 17^th^ Place

Mail Stop B119

Aurora, CO 80045

USA

^d^Department of Pediatrics, College of Medicine, The Ohio State University

370 W. 9^th^ Avenue

Columbus, OH 43210

USA

Corresponding Author: Motao Zhu

700 Children’s Drive, RB3-WB5217,

Columbus, OH 43205

USA

Phone: +1 (614) 355-6687

Email: [Motao.Zhu@NationwideChildrens.org](mailto:Motao.Zhu@NationwideChildrens.org)

Author Emails:

Marco.Benedetti@nationwidechildrens.org

Li.6043@osu.edu

Lucas.Neuroth@nationwidechildrens.org

Kayleigh.Humphries@nationwidechildrens.org

Ashley.Brooks-Russell@cuanschutz.edu

Motao.Zhu@nationwidechildrens.org

In this document, we present a set of tables to supplement the manuscript *Demographic and policy-based differences in behaviors and attitudes towards driving after marijuana use: an analysis of the 2013-2017 Traffic Safety Culture Index.* Tables S1 and S2 tabulate the effective dates of recreational, medical, and per-se marijuana policies that were used in the main analysis. Table S3 presents the results from the main analysis, however the *p-*values are not corrected for multiple comparisons, as they were in the manuscript. Finally, Table S4 presents policy-based differences in the three outcomes only among those who self-reported using marijuana.

| **State** | **Effective date of recreational marijuana legalization** | **Effective date of medical marijuana legalization** | **Effective date of per-se marijuana law** |
| --- | --- | --- | --- |
| Alabama | **--** | **--** | **--** |
| Alaska | 2/24/2015 | 3/4/1999 | **--** |
| Arizona | **--** | 4/14/2011 | **--** |
| Arkansas | **--** | 11/9/2016 | **--** |
| California | 11/9/2016 | 11/6/1996 | **--** |
| Colorado | 12/10/2012 | 12/28/2000 | **--** |
| Connecticut | **--** | 5/31/2012 | **--** |
| Delaware | **--** | 7/1/2011 | 6/28/2006 |
| District of Columbia | 2/26/2015 | 2/25/2010 | **--** |
| Florida | **--** | 1/3/2017 | **--** |
| Georgia | **--** | **--** | 1/1/2000 |
| Hawaii | **--** | 6/14/2000 | **--** |
| Idaho | **--** | **--** | **--** |
| Illinois | 6/25/2019 | 1/1/2014 | 7/29/2016 |
| Indiana | **--** | **--** | 1/1/2000 |
| Iowa | **--** | **--** | 7/1/1997 |
| Kansas | **--** | **--** | **--** |
| Kentucky | **--** | **--** | **--** |
| Louisiana | **--** | 6/29/2015 | **--** |
| Maine | 1/30/2017 | 12/23/1999 | **--** |
| Maryland |  | 6/1/2014 | **--** |
| Massachusetts | 12/15/2016 | 1/1/2013 | **--** |
| Michigan | 12/6/2018 | 12/4/2008 | 7/15/2003 |
| Minnesota | **--** | 5/30/2014 | **--** |
| Mississippi | **--** | **--** | **--** |

**Table S1: Effective dates of state marijuana policies: Alabama-Mississippi**

| **State** | **Effective date of recreational marijuana legalization** | **Effective date of medical marijuana legalization** | **Effective date of per-se marijuana law** |
| --- | --- | --- | --- |
| Missouri | **--** | 12/6/2018 | **--** |
| Montana | **--** | 11/2/2004 | 10/1/2013 |
| Nebraska | **--** | **--** | **--** |
| Nevada | 1/1/2017 | 10/1/2001 | 6/11/1999 |
| New Hampshire | **--** | 7/23/2013 | **--** |
| New Jersey | **--** | 10/1/2010 | **--** |
| New Mexico | **--** | 7/1/2007 | **--** |
| New York | **--** | 7/5/2014 | **--** |
| North Carolina | **--** | **--** | **--** |
| North Dakota | **--** | 4/18/2017 | **--** |
| Ohio | **--** | 9/8/2016 | 8/17/2006 |
| Oklahoma | **--** | 7/26/2018 | 10/1/2013 |
| Oregon | 6/30/2015 | 1/1/1998 | **--** |
| Pennsylvania | **--** | 5/18/2016 | 2/1/2004 |
| Rhode Island | **--** | 1/3/2006 | 7/12/1990 |
| South Carolina | **--** | **--** | **--** |
| South Dakota | **--** | **--** | **--** |
| Tennessee | **--** | **--** | **--** |
| Texas | **--** | **--** | **--** |
| Utah | **--** | 12/1/2018 | 5/2/1994 |
| Vermont | 7/1/2018 | 5/26/2004 | **--** |
| Virginia | **--** | **--** | **--** |
| Washington | 12/6/2012 | 11/3/1998 | 12/6/2012 |
| West Virginia | **--** | 7/5/2017 | 7/5/2017 |
| Wisconsin | **--** | **--** | 12/19/2003 |
| Wyoming | **--** | **--** | **--** |

**Table S2: Effective dates of state marijuana policies: Missouri-Wyoming**

| **Variable** | **Level** | **Unweighted sample size**^a^ | **Weighted percent of total sample**  **(95% CI)** | **Drivers who self-reported driving within one hour of using marijuana at least once in the last year.** | | **Respondents who said driving within one hour of using marijuana is somewhat or completely acceptable.** | | **Respondents who somewhat or strongly support a per-se marijuana law.** | |
| --- | --- | --- | --- | --- | --- | --- | --- | --- | --- |
|  |  |  |  | **Weighted**  **percent**  **(95% CI)** | **Rao-Scott** $\boldsymbol{\chi}^{\boldsymbol{2}}$ **p-value**^b^ | **Weighted**  **percent**  **(95% CI)** | **Rao-Scott** $\boldsymbol{\chi}^{\boldsymbol{2}}$ **p-value**^b^ | **Weighted**  **percent**  **(95% CI)** | **Rao-Scott** $\boldsymbol{\chi}^{\boldsymbol{2}}$ **p-value**^b^ |
| Full Sample | NA | 11,816 | -- | 5.0 (4.5, 5.4) | -- | 9.7 (9.1, 10.3) | -- | 82.6 (81.8, 83.4) | -- |
| Gender | Male | 5,753 | 48.1 (47.1, 49.2) | 6.5 (5.8, 7.3) |  | 11.1 (10.1, 12.0) |  | 80.5 (79.2, 81.7) |  |
|  | Female | 6,063 | 51.9 (50.8, 52.9) | 3.5 (2.9, 4.1) | **<0.001** | 8.4 (7.6, 9.3) | **<0.001** | 84.4 (83.4, 85.5) | **<0.001** |
| Income | <$25,000 | 1,888 | 17.3 (16.5, 18.1) | 9.4 (7.6, 11.2) |  | 13.8 (12.0, 15.7) |  | 76.0 (73.7, 78.3) |  |
|  | $25,000-$49,999 | 2,570 | 22.0 (21.1, 22.9) | 5.7 (4.5, 6.8) |  | 10.1 (8.8, 11.5) |  | 81.6 (79.8, 83.4) |  |
|  | $50,000-$74,999 | 2,220 | 18.2 (17.5, 19.0) | 4.3 (3.2, 5.3) |  | 9.2 (7.8, 10.7) |  | 83.9 (82.1, 85.7) |  |
|  | $75,000-$99,999 | 1,708 | 14.6 (13.9, 15.4) | 3.8 (2.7, 4.9) |  | 9.3 (7.6, 11.0) |  | 82.9 (80.8, 85.0) |  |
|  | $100,000 or more | 3430 | 27.8 (26.9, 28.7) | 3.5 (2.8, 4.2) | **<0.001** | 7.3 (6.3, 8.3) | **<0.001** | 86.2 (84.8, 87.5) | **<0.001** |
| Race/ ethnicity | Non-Hispanic White | 8,696 | 66.0 (65.0, 67.1) | 4.3 (3.8, 4.9) |  | 9.9 (9.1, 10.6) |  | 83.1 (82.1, 84.0) |  |
|  | Non-Hispanic Black | 1,047 | 11.5 (10.8, 12.3) | 7.5 (5.5, 9.5) |  | 11.5 (9.2, 13.7) |  | 76.5 (73.5, 79.5) |  |
|  | Non-Hispanic Other | 409 | 5.0 (4.4, 5.5) | 5.9 (3.1, 8.7) |  | 9.3 (6.1, 12.5) |  | 89.5 (86.3, 92.6) |  |
|  | Hispanic | 1,319 | 15.0 (14.1, 15.8) | 5.2 (3.7, 6.7) |  | 7.7 (6.0, 9.4) |  | 83.9 (81.6, 86.2) |  |
|  | Non-Hispanic 2+ races | 345 | 2.5 (2.2, 2.9) | 10.1 (5.3, 14.8) | **<0.001** | 9.6 (6.1, 13.2) | 0.080 | 74.6 (68.9, 80.3) | **<0.001** |
| Education | Less than high school | 1,060 | 12.4 (11.6, 13.1) | 8.5 (6.3, 10.7) |  | 11.0 (8.9, 13.2) |  | 78.1 (75.3, 80.9) |  |
|  | High school | 3,448 | 28.7 (27.7, 29.6) | 4.7 (3.8, 5.5) |  | 9.7 (8.6, 10.9) |  | 82.0 (80.5, 83.5) |  |
|  | Some college | 3,389 | 29.7 (28.8, 30.7) | 5.5 (4.5, 6.4) |  | 11.1 (9.9, 12.4) |  | 81.4 (79.9, 83.0) |  |
|  | Bachelor’s or higher | 3,919 | 29.2 (28.3, 30.2) | 3.6 (2.9, 4.3) | **<0.001** | 7.6 (6.6, 8.6) | **<0.001** | 86.0 (84.7, 87.3) | **<0.001** |
| Age | 19-29 | 1,870 | 19.7 (18.8, 20.6) | 11.6 (9.7, 13.4) |  | 15.1 (13.3, 17.0) |  | 76.0 (73.7, 78.3) |  |
|  | 30-44 | 2,661 | 25.7 (24.7, 26.6) | 5.6 (4.6, 6.5) |  | 12.0 (10.6, 13.5) |  | 79.8 (78.1, 81.6) |  |
|  | 45-59 | 3,608 | 27.3 (26.4, 28.2) | 3.4 (2.7, 4.1) |  | 8.8 (7.7, 9.8) |  | 83.4 (82.0, 84.8) |  |
|  | 60 or older | 3,677 | 27.4 (26.5, 28.3) | 1.8 (1.3, 2.3) | **<0.001** | 4.6 (3.8, 5.4) | **<0.001** | 88.7 (87.5, 89.9) | **<0.001** |
| Marijuana Policy | RM and MM illegal | 6,011 | 51.5 (50.4, 52.5) | 4.3 (3.7, 5.0) |  | 9.6 (8.7, 10.5) |  | 81.7 (80.5, 82.8) |  |
|  | RM illegal, MM legal | 4855 | 41.0 (40.0, 42.0) | 5.9 (5.1, 6.8) |  | 9.6 (8.6, 10.6) |  | 82.6 (81.4, 83.9) |  |
|  | RM and MM legal | 950 | 7.5 (7.0,8.1) | 4.2 (2.7, 5.8) | **0.005** | 10.6 (8.2,13.0) | 0.733 | 87.8 (85.3, 90.3) | **0.001** |
| Per-se law | Absent | 8,769 | 72.8 (71.9, 73.8) | 5.1 (4.6, 5.7) |  | 10.0 (9.3, 10.8) |  | 81.6 (80.6, 82.5) |  |
|  | Present | 3,047 | 27.2 (26.2, 28.1) | 4.5 (3.6, 5.4) | 0.282 | 8.8 (7.6, 9.9) | 0.086 | 85.1 (83.6, 85.5) | **<0.001** |

**Table S3: 2013-2017 TSCI estimated past-year driving within one hour of marijuana use, personal acceptance of driving after marijuana use, and support for per-se laws with unadjusted p-values.**

^a^The unweighted sample size includes those with missing values or non-response to questions (1)-(3).

^b^Boldface denotes statistical significance at the threshold of 0.05.

| **Variable** | **Level** | **Unweighted sample size**^a^ | **Weighted percent of total sample**  **(95% CI)** | **Marijuana users who self-reported driving within one hour of using marijuana at least once in the last year.** | | **Marijuana users who said driving within one hour of using marijuana is somewhat or completely acceptable.** | | **Marijuana users who somewhat or strongly support a per-se marijuana law.** | |
| --- | --- | --- | --- | --- | --- | --- | --- | --- | --- |
|  |  |  |  | **Weighted**  **percent**  **(95% CI)** | **Rao-Scott** $\boldsymbol{\chi}^{\boldsymbol{2}}$ **p-value**^b^ | **Weighted**  **percent**  **(95% CI)** | **Rao-Scott** $\boldsymbol{\chi}^{\boldsymbol{2}}$ **p-value**^b^ | **Weighted**  **percent**  **(95% CI)** | **Rao-Scott** $\boldsymbol{\chi}^{\boldsymbol{2}}$ **p-value**^b^ |
| Full Sample | NA | 1,131 | -- | 46.3 (42.5, 50.1) | -- | 39.1 (35.3, 42.8) | -- | 53.2 (49.4, 57.0) | -- |
| Marijuana Policy | RM and MM illegal | 501 | 45.0 (42.4, 47.5) | 48.9 (43.7, 54.1) |  | 43.9 (38.1, 49.7) |  | 48.0 (42.2, 53.8) |  |
|  | RM illegal, MM legal | 502 | 44.8 (41.9, 47.7) | 50.6 (45.5, 55.8) |  | 36.9 (31.4, 42.3) |  | 54.3 (48.6, 59.9) |  |
|  | RM and MM legal | 128 | 10.2 (8.5, 11.9) | 28.0 (19.0, 37.0) | **<0.001** | 29.2 (18.8, 39.2) | **0.035** | 68.9 (58.5, 79.4) | **0.004** |
| Per-se law | Absent | 851 | 73.8 (71.3, 76.3) | 48.0 (44.0, 51.9) |  | 39.8 (35.5, 44.1) |  | 51.1 (46.8, 55.5) |  |
|  | Present | 280 | 26.2 (23.7, 28.6) | 45.7 (38.9, 52.4) | 0.562 | 37.1 (29.8, 44.4) | 0.539 | 58.9 (51.5, 66.4) | 0.079 |

**Table S4: 2013-2017 TSCI estimated past-year driving within one hour of marijuana use, personal acceptance of driving after marijuana use, and support for per-se laws among respondents who self-reported using marijuana in the last year.**

^a^The unweighted sample size includes those with missing values or non-response to questions (1)-(3).

^b^Boldface denotes statistical significance at the threshold of 0.05.
